# Rethinking the Blue Economy: Integrating social science for sustainability and justice

**DOI:** 10.1038/s44183-025-00138-1

**Published:** 2025-07-12

**Authors:** Jerneja Penca, Irmak Ertör, Marta Ballesteros, Michael Briguglio, Maciej Kowalewski, Birgit Pauksztat, Dražen Cepić, Cristina Piñeiro-Corbeira, Natasa Vaidianu, Sebastian Villasante, José J. Pascual-Fernández

**Affiliations:** 1 Science and Research Centre Koper, Mediterranean Institute for Environmental Studies, Koper, Slovenia; 2 Bogazici University, The Ataturk Institute for Modern Turkish History, Istanbul, Turkey; 3 Spanish Institute of Oceanography (IEO-CSIC), Vigo, Spain; 4 University of Malta, Msida, Malta; 5 University of Szczecin, Institute of Sociology, UNESCO Chair for Social Sustainability, Szczecin, Poland; 6 Nordland Research Institute, Bodø, Norway; 7 Institute for Social Research in Zagreb, Zagreb, Croatia; 8 Universidad de A Coruña, BioCost Research Group, Centro Interdisciplinar de Química e Bioloxía (CICA), Facultad de Ciencias, A Coruña, Spain; 9 Ovidius University of Constanta, Faculty of Natural Sciences and Agricultural Sciences, Constanța, Romania; 10 University of Bucharest, CICADIT Center, Bucharest, Romania; 11 University of Santiago de Compostela, EqualSea Lab-CRETUS, A Coruña, Spain; 12 Universidad de La Laguna, Instituto Universitario de Investigación Social y Turismo, Tenerife, Spain

**Keywords:** Climate-change adaptation, Climate-change policy, Sustainability, Scientific community, Social sciences, Environmental studies

## Abstract

To fulfill the Blue Economy’s promise of sustainable and just ocean use, its scientific foundation must more fully integrate the social sciences. Drawing on insights from real-world scientific networking initiatives, we identify three key contributions of the social sciences and propose a strategy to redefine the Blue Economy. This strategy anchors knowledge in societal challenges and emphasizes co-creation, the science-policy interface, knowledge integration, and the values of accountability and care.

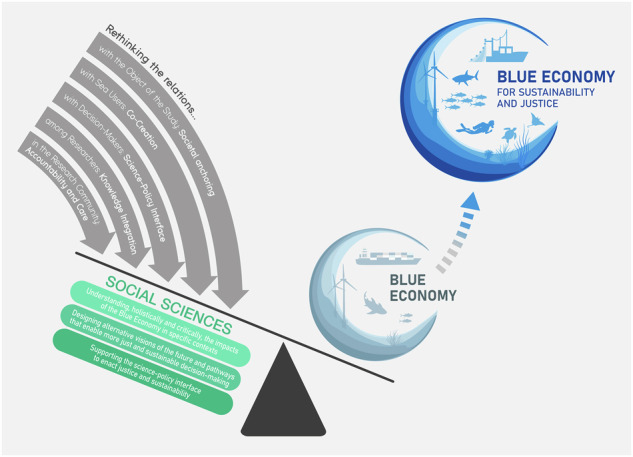

## Introduction

When the term Blue Economy was coined in 2011 by the Small Island Developing States (SIDS), it offered a new focus on the seas. It encouraged viewing the ocean as a space, as a living entity, and as a resource base capable of promoting the prosperity of people, especially those who live with and off it^1^. This concept was a strong call for innovation to reduce inequalities and minimize the environmental impact of traditional (e.g. fisheries and aquaculture) and new economic activities (e.g. biotechnology, wind energy and deep-sea mining). With the seas and oceans becoming increasingly important in geopolitics and economics, it was essentially an appeal to “do things differently” at sea and to learn from what has been done on land.

Despite the remarkable agency of SIDS in shaping the discourse of the Blue Economy towards social equity, sustainable livelihoods, and ocean stewardship^2–5^, that innovative, emancipatory, and ecosystem-based potential of the concept seems today unlikely to materialize. While the Blue Economy continues to be invoked as both a descriptive term (referring to the activities of industries related to ocean environments, along with the assets, goods, and services provided by marine ecosystems)^6^ and a signpost for action (aiming at sustainable, resilient, and equitable activities at sea)^7–9^, attempts to implement the Blue Economy are resulting in unintended negative impacts on the environment and people. This is turning some of the early concerns about the concept into reality^10^ and transforming the inherently interdependent and reciprocal relationship between humans and oceans^11,12^ into an extractive and adversarial dynamic. For example, the rapid expansion of the cruise industry, promoted as a promising sector in the early enthusiasm for the ocean-based economic development^13–15^, is now widely recognized as adversely affecting water, air, and land ecosystems, including non-human species, as well as the health of passengers, crew, and residents in cruise ports^16^. Similarly, the growth of offshore wind farms, solar photovoltaics, and other renewable energy projects—driven by the global push for sustainable, cost-effective energy alternatives to both fossil and non-fossil fuels^17^—raises concerns about the displacement of other marine activities, such as fishing or aquaculture^7,18^.

The Blue Economy, as it is implemented today, is contributing to a wide range of injustices and inequalities^19,20^, as well as a disproportionate concentration of capital and resources in a few dominant actors^21,22^. It also leads to the intensification of pollution and environmental pressures in the ocean^23^, while it has not eliminated hazardous working conditions and breaches of human rights^24^ for maritime workers and fishers^25,26^, or reduced exclusion of indigenous peoples and women^27^. Further, the expansion of the Blue Economy that demands more goods and services from the ocean could lead to an increase in conflicts^2,28^. Paradoxically, a concept originally devised to empower the powerless appears to have been co-opted and leveraged mostly by the rich and powerful^29^.

This uneven and disappointing implementation of the Blue Economy concept has translated into various responses on the scientific and policy levels, calling for more actionable^30,31^, better-organized and coordinated^32,33^, and more diverse and equitable science^34,35^. Such calls are often accompanied by abandoning the Blue Economy discourse in favour of alternative frameworks, such as Blue Justice, Ocean Health, or Ocean Sustainability that bring much-needed attention to equity, ecological integrity, and long-term stewardship. While these alternative framings address critical issues, they can inadvertently widen the gap between science and policy. The pursuit of conceptual precision and accuracy through new concepts, however, can create a disconnect between critical academic debates and the continuing policy implementation of the Blue Economy. Parallel discourses can make consensus on Blue Economy more difficult and narrow the scope of the scientific community’s influence on policymaking, leaving policymakers ill-equipped to draw on (a full spectrum of) scientific findings to inform and improve decision-making, including in ongoing interfaces that seek to integrate scientific knowledge into sustainable ocean governance, such as the creation of Blue Economy fora^36^, the UN Decade of Ocean Science for Sustainable Development^37^, the High-Level Panel for a Sustainable Ocean Economy^38^, or the initiative to establish an International Panel for Ocean Sustainability^39^. This fragmentation of policy-relevant knowledge weakens the societal ability to withstand the intensifying geopolitical and exploitative pressures^40^ on the ocean, by moving away from further economic growth and exploitation towards sustainable and equitable pathways. It also diminishes the relevance of social science to the emerging and forthcoming uses of the marine space, as well as its ability to shape scientific and public debates, actions, and outcomes^41,42^.

The Blue Economy concept has demonstrated an appeal for policy discourse and the private sector. It is easily understandable, partly because of its versatile and ambiguous meaning, which shapes funding partnerships^43,44^ and policy initiatives^45^. While the Blue Economy concept is contested for its depoliticizing effect and hegemonic status^46^, it has an unequivocal integrative potential. In this paper, we argue for the need for a critical revision of the Blue Economy discourse rather than its abandonment. We work with those who have used this concept effectively to facilitate the bridging of the often-challenging science-policy divide^47–49^. However, we argue that to promote better-informed decision-making and the implementation of the Blue Economy in a way that is more just and sustainable, improved production of knowledge about the Blue Economy is necessary. One crucial aspect of this is the need to more fully incorporate analytical perspectives from the social sciences.

We present a roadmap for the future that includes a better integration of the social sciences in the creation of marine and maritime knowledge and in its exchange with other societal domains. We integrate prior calls for using social knowledge for ocean sustainability^50,51^ with our own reflexive approach to constructing research findings^52^ and sharing them^53^. In subsequent section, we explain *why* it is necessary to fully incorporate the social sciences into the Blue Economy. Then, we present *how* this can be done by the scientific community, by outlining the rationale and functioning of actual effort.

## Rethinking the Blue Economy with the role of the social sciences

The social sciences are instrumental in building a more just and sustainable Blue Economy through three broad contributions: (a) *understanding, holistically and critically, the impacts of the Blue Economy* in specific contexts and identifying power asymmetries and injustices; (b) *designing alternative visions of the future and pathways* that enable more just and sustainable decision-making; and (c) *supporting the science-policy interface to enact justice and sustainability*. All of these bring direct benefits to decision-makers.

Firstly, it is only through in-depth social science research that we can fully comprehend the socio-ecological dimensions in which the Blue Economy operates. The social sciences enable us to appreciate complexities of time and space, identify injustices and inequities in the outcomes of Blue Economy initiatives, assess their varied impacts on different societal groups, and uncover the cultural, technological, political, and economic dynamics and systemic forces that shape them. One example would be the Small-Scale Fisheries (SSF). The social sciences have been vital in pointing out the immense contribution of SSF to supplying nutritious and healthy food and livelihoods worldwide^54,55^, identifying a wide range of injustices and inequities to which SSF are subjected, and explaining the reasons for and impacts of these^56,57^. Decision-makers can benefit directly from the identification of specific barriers that SSF face in accessing seafood markets^58,59^, obtaining fishing rights^60^, or accessing fishing grounds^61^. As such, they can replace the dysfunctional systemic prioritization of more powerful actors.

Secondly, social science frameworks are essential for designing alternative visions of the future and pathways that promote greater sustainability and justice. The social sciences’ commitment to highlighting diverse perspectives and worldviews not only supports informed and legitimate decision-making but also plays a critical role in challenging dominant paradigms and proposing transformative alternatives^62^. This is particularly important when visioning involves epistemologically marginalized or historically disadvantaged groups, such as indigenous peoples and local communities^63^. One example of the importance of discovering alternative visions is Maritime Spatial Planning (MSP). MSP promises to coordinate an increasing number of activities in shared spaces in order to avoid conflicts^64,65^. However, this process or tool is not inherently impartial and rational, and it is not unusual to see it conducted in a way that ignores traditional activities at sea^66,67^. Social science research has developed practices in MSP that challenge dominant policy goals and approaches and offer more open-minded and inclusive ways of shaping the future use of maritime space^68,69^. While not disregarding the political considerations and power dynamics of the MSP^70^ or any other process^71,72^, the social sciences can open the eyes of decision-makers towards alternative goals and approaches in creating a more equitable management, governance, and cohabitation of the seas.

Thirdly, the social sciences can actively support the science-society-policy interface to enact sustainability and justice. Social scientists can design conceptual ‘boundary objects’ that generate shared understandings and facilitate interactions between different actors, perspectives, and types of knowledge^73,74^. They can organize participatory processes for inclusive and collaborative engagement with and for communities, and propose holistic and operational decision-making tools^75–77^. A case in point is the creation of Marine Protected Areas, where social scientists have helped with the conception of the sites, facilitated communication and discussion among stakeholders in drafting the management plan and supported its monitoring^78,79^. Decision-makers do well to include social scientists when conducting any participatory campaigns in order to avoid ineffective policies or having them challenged in the courts, and to create more robust and lasting policies.

## The strategy for rethinking the Blue Economy

With a stronger emphasis on social science, Rethinking the Blue Economy calls for the re-organization of the scientific community both internally and in how it engages with broader societal and political realms. Reflexivity – at both the individual and group levels – is a necessary condition for any meaningful reform. To illustrate how such a shift can take shape, we draw on several real-world networking initiatives that work to link scientific knowledge with governance processes. The most recent one is the COST Action Rethinking the Blue Economy: Socio-ecological impacts and opportunities (RethinkBlue), which builds on a range of previous collaborations and interacts with other parallel initiatives, e.g. COST Action Ocean Governance for Sustainability – challenges, options and the role of science (OceanGov), biannual MARE People & the Sea conferences, the Programme on Ecosystem Change and Society (PECS) conferences, One Ocean Hub, and others. From these, we distil five organizing principles that offer concrete guidance for reimagining the Blue Economy. We intentionally prioritize lived experiences over theoretical frameworks in order to move beyond an abstract critique of the scientific community and towards a grounded, adaptive strategy for practicing it, also highlighting that change is already being tested, iterated, and advanced.

### Rethinking the relations with the object of the study: societal anchoring

The Blue Economy should be re-oriented from economic sectors as traditionally articulated (fisheries, transport, energy, etc.) towards themes that are anchored in human and societal challenges, concerns and aspirations. For example, inquiries (or working groups) can be dedicated to maritime occupations, food security and sustainable consumption, port cities and coastal communities, fisheries governance and emergent activities, or climate change and natural hazards. Such human-centric approach is more likely to acknowledge the complex causal interrelations among sectors^80^ and inherently trigger non-siloed insights and collaboration beyond the traditional communities (e.g. when examining the impact of climate change on fisheries and the consumption of seafood).

### Rethinking the relations with sea users: co-creation

The production of knowledge on issues such as climate impacts, new technologies or shifts in value chains should move from being intra-academic to being co-produced and co-created in transdisciplinary research with stakeholders beyond academia. Knowledge co-production involves stakeholders both in decisions and in identifying shared goals^81^. A pilot initiative demonstrates the value of this in practice: fishing organizations, public institutions, and civil society groups collaborated in supplying local fish to school canteens as alternatives to markets in enhancing sustainability and promoting local consumption^82^. While such locally co-created knowledge is context-specific, it can also be combined with experience from other regions and countries – either to highlight diversity or shared societal effects^58^. In a network, transnational studies, policy briefs, and position statements with more conceptual and transnational findings can feed into strategies or shared policy frameworks.

### Rethinking the relations with decision-makers: science-policy interface

Rethinking the Blue Economy should involve active engagement in the science-policy interface, i.e. the processes and activities that connect different knowledge domains and organizations, facilitating the exchange of information and ideas between them to ensure that scientific evidence effectively informs decision-making processes^83^. Scholars should not shy away from engaging in it, targeting in particular the policy level that is most relevant, e.g. the EU-level, or the global fisheries industry. Formats, such as policy briefs that summarize research publications in accessible formats, trainings, webinars and presentations at policy-relevant events, are some of the possible tools to this end.

### Rethinking the relations among researchers: knowledge integration

The building of a knowledge base should move from maintaining established disciplines, themes, and stable communities to integrating knowledge and improving the interaction among participating scientists. Active maintenance of inclusive networks is a primary tool for that, with a focus on new collaborations, working group meetings, training courses, mentorship programmes, research visits, conferences, and the regular communication of news and opportunities. In practising inclusiveness, attention should be paid to overcoming existing fragmentations and marginalizations. These can be related to researchers’ skills, career stage, access to research funding and infrastructure, or the status of innovators from non-academic institutions or countries with lower levels of research intensity. Addressing these fragmentations also means involving, in the research, profiles that are particularly marginalized in the Blue Economy — such as historians, landscape architects, and user interface designers — for example, by actively integrating them as speakers in seminar series. Further, constant attention should be paid to ensuring that topic framings emerging from certain cultures or countries do not become hegemonic in the research discourse.

### Rethinking the relations in the research community: accountability and care

Commitment to ensuring pleasant relations and ethical operation of structures is a key. Clear governance structures and practices should operate by consensual decision-making and transparent reporting to the members and stakeholders. These structures should be accompanied by a value-set centring on accountability, dialogue, mutual respect, care, and kindness that impact on developing specific actions. Rethinking the Blue Economy provides a direct opportunity to put into action a different way of running our lives and work by making kind and caring relationships central^84^. Insofar as relational emphasis counters time efficiency, teamwork (e.g. in leading activities) becomes an important aspect of such collaboration, understood as the division of work and the provision of opportunities for discussion, feedback, and an incremental development of leadership skills for less experienced collaborators.

## Rethinking the Blue Economy – Collectively

Rethinking the Blue Economy as a framework that prioritizes sustainability and justice does not require the disengagement of social scientists, but rather a more vocal integration of their questions and methodologies into transdisciplinary efforts, alongside the promotion of more relational principles. Rethinking the Blue Economy is not a task for the scientific community alone: it also calls for decision-makers to adopt revised metrics and assessment tools to reshape governance priorities toward broader societal goals of well-being, equity, and sustainability, alongside new governance models, built on collaboration and the inclusion of diverse stakeholders’ perspectives and knowledge.

Across various contexts, opportunities and initiatives are emerging that are rooted in sustainability, justice and systemic reimagining, often of non-linear, interconnected character. Rethinking the Blue Economy is necessary for all who recognize the urgency of change and the power of collective action to make it possible.

## Data Availability

Data sharing is not applicable to this article as no datasets were generated or analysed during the current study.
